# Recent advances in the management of pulmonary arterial hypertension

**DOI:** 10.12688/f1000research.9739.1

**Published:** 2016-11-24

**Authors:** Halley Tsai, Yon K. Sung, Vinicio de Jesus Perez

**Affiliations:** 1 Division of Pulmonary/Critical Care, Stanford University School of Medicine, Stanford, CA, 94305-5236, USA; 2 Vera Moulton Wall Center for Pulmonary Vascular Disease, Stanford University School of Medicine, Stanford, CA, 94305-5414, USA

**Keywords:** pulmonary arterial hypertension, riociguat, macitentan, treprostinil, selexipag

## Abstract

Over the past 20 years, there has been an explosion in the development of therapeutics to treat pulmonary arterial hypertension (PAH), a rare but life-threatening disorder associated with progressive elevation of pulmonary pressures and severe right heart failure. Recently, the field has seen the introduction of riociguat, a soluble guanylate cyclase stimulator, a new endothelin receptor antagonist (macitentan), and oral prostanoids (treprostinil and selexipag). Besides new drugs, there have been significant advances in defining the role of upfront combination therapy in treatment-naïve patients as well as proposed methods to deliver systemic prostanoids by use of implantable pumps. In this review, we will touch upon the most important developments in PAH therapeutics over the last three years and how these have changed the guidelines for the treatment of PAH. These exciting developments herald a new era in the treatment of PAH which will be punctuated by the use of more clinically relevant endpoints in clinical research trials and a novel treatment paradigm that may involve upfront double- or triple-combination therapy. We anticipate that the future will make use of these strategies to test the efficacy of upcoming new drugs that aspire to reduce disease progression and improve survival in patients afflicted with this devastating disease.

## Introduction

Pulmonary arterial hypertension (PAH) is a life-threatening disease associated with progressive elevation of pulmonary pressures that leads to right heart failure and death ^1^. PAH is a rare disease with an estimated prevalence of about 15 cases per million patients ^2^. The diagnosis is based on pressure measurements obtained by right heart catheterization and is defined as a mean pulmonary artery pressure of at least 25 mmHg, a pulmonary artery wedge pressure of not more than 15 mmHg, and a pulmonary vascular resistance (PVR) of at least 3 Wood units. PAH is characterized by remodeling and progressive loss of the small- to medium-sized pulmonary arterioles with eccentric and obliterative thickening of the intima and media, composed mainly of smooth muscle cells and myofibroblasts. The hallmark of PAH is the plexiform lesion, a disorganized growth of endothelial cells that form false channels and thereby prevent blood flow to the capillaries ^3^. The inciting event for these pathological changes is thought to be a combination of genetic and environmental insults that trigger endothelial cell injury. This, coupled with impaired vascular regeneration, leads to progressive loss of small pulmonary arteries ^4, 5^.

**Figure 1.**
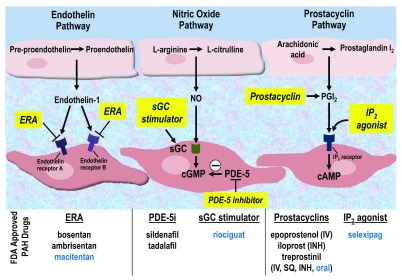
Pathways targeted in current therapies for pulmonary arterial hypertension. Newly approved therapies are listed in blue. cAMP, cyclic adenosine monophosphate; cGMP, cyclic guanylate monophosphate; ERA, endothelin receptor antagonist; FDA, US Food and Drug Administration; INH, inhaled; IP _2_, prostacyclin receptor 2; IV, intravenous; NO, nitric oxide; PAH, pulmonary arterial hypertension; PDE-5, phosphodiesterase-5; PDE-5i, phosphodiesterase-5 inhibitor; PGI _2_, prostaglandin I _2_; sGC, soluble guanylate cyclase; SQ, subcutaneous.

Prior to the approval of epoprostenol in 1995 ^6^, there were no specific therapies for PAH and survival was very poor; 1-year survival was 69%, and 5-year survival was only 38% ^7^. In subsequent years, the rapid development of new medications for PAH has resulted in improved patient outcomes as documented by the REVEAL (Registry to Evaluate Early and Long-term PAH Disease Management) and French Consortium registries ^8– 11^. These therapies, which are all primarily pulmonary vasodilators, target one of three pathways: (1) nitric oxide (NO), (2) endothelin, and (3) prostaglandin pathways ( Figure 1). Historically, the NO pathway has been targeted by phosphodiesterase-5 inhibitors (PDE-5is), the endothelin pathway by endothelin receptor antagonists (ERAs), and the prostaglandin pathway by prostacyclin analogues.

Over the past few years, there have been a number of advances in the management of PAH, which will be discussed in this review. In the past three years alone, four new oral medications have been approved for the treatment of PAH; these medications are also pulmonary vasodilators that target one of the three pathways listed above, although two have novel mechanisms of action. In addition to new medications, different treatment strategies and different modes of medication delivery have been explored. Finally, it is important to point out that use of non-pharmacological interventions like participation in exercise training programs has been advocated for the patient with PAH as they have been shown to increase exercise capacity, pulmonary hemodynamics, and health-related quality of life ^12^. This is relevant in light of recent studies that have shown atrophy and dysfunction of striated muscles associated with the respiratory and musculoskeletal systems ^13^.

## Newly approved medications for pulmonary arterial hypertension

### Riociguat

As the endothelial dysfunction associated with the development of PAH results in decreased production of the endogenous vasodilatory mediators NO and downstream cyclic guanylate monophosphate (cGMP), modulators of the NO pathway continue to be investigated. Riociguat is a novel compound that acts on the NO pathway by stimulating soluble guanylate cyclase (sGC). Unlike PDE-5is, which prevent the breakdown of cGMP, riociguat promotes the production of cGMP by both sensitizing sGC to endogenous NO and directly stimulating sGC independently of NO ^14^.

The efficacy of riociguat was tested in the PATENT-1 (PAH Soluble Guanylate Cyclase-Stimulator Trial 1) and PATENT-2 trials. PATENT-1 was a multicenter, randomized, double-blind, placebo-controlled trial of riociguat in 443 World Health Organization (WHO) group 1 PAH patients with or without background therapy with ERAs or prostacyclins or both ^15^. Background PAH therapy did not include a PDE-5i or other stimulators of the NO pathway. This study found that riociguat was beneficial for PAH patients regardless of underlying functional class (FC) or background therapy, as demonstrated by a statistically significant increase in 6-minute walk distance (6MWD) of 30 m in the treatment group compared with a decrease of 6 m in the placebo group at 12 weeks ( *P* ≤0.001). The active drug group also had improvements in secondary endpoints, including PVR, N-terminal pro-brain natriuretic peptide (NT-proBNP), FC, and time to clinical worsening (TTCW) ^15^. Riociguat was approved by the US Food and Drug Administration (FDA) for the treatment of WHO group 1 PAH in October 2013. PATENT-2, the long-term open-label extension trial, enrolled 396 of the 443 patients from PATENT-1, and all patients were transitioned to active treatment with riociguat at 2.5 mg three times per day ^16^. Trial investigators found that the improvements in 6MWD and WHO FC were maintained for up to 1 year. Compared with PATENT-1 baseline values, the 6MWD improved by 51 ± 74 m and the WHO FC improved in 33% of the patients.

In 2015, the PATENT PLUS study, a blinded, randomized, extension study of riociguat in PAH, evaluated the safety and efficacy of riociguat in combination with the PDE-5i, sildenafil ^17^. A total of 18 PAH patients receiving sildenafil 20 mg three times per day were randomly assigned to placebo or riociguat for 12 weeks. There was no significant clinical benefit to combination riociguat/sildenafil therapy, and long-term follow-up (mean total treatment duration of 305 days) showed higher rates of discontinuation of therapy in the combination arm because of hypotension. Although the study was small, the lack of a positive risk-benefit ratio and potential for adverse long-term effects concluded that concomitant use of riociguat with a PDE-5i is contraindicated.

### Macitentan

The SERAPHIN (Study with an Endothelin Receptor Antagonist in PAH to Improve Clinical Outcomes) study was a randomized, double-blind, placebo-controlled, phase 3 trial designed to test macitentan, a once-daily oral dual endothelin A/B (ETA/ETB) receptor antagonist that exhibits high binding affinity to ETA and greater tissue penetration than the parent molecule, bosentan ^18^. The primary endpoints of SERAPHIN, in contrast to those of prior studies with ERAs which have looked at changes in 6MWD at short time intervals (12 to 16 weeks), were morbidity and mortality measured through a composite TTCW event, defined as worsening PAH, initiation of parenteral prostanoids, lung transplantation, atrial septostomy, or death. Changes in exercise capacity, FC, and hemodynamics were collected as secondary endpoints. In total, 742 predominantly WHO FC II and III patients were recruited from 151 centers in 39 countries and randomly assigned to receive placebo (n = 250) or macitentan 3 mg (n = 250) or 10 mg (n = 242). Most patients (64%) were on background therapy with PDE-5i or prostanoids or both, whereas 36% were treatment naïve at baseline. Over a period of 115 weeks, the primary endpoint occurred in 46.4% of patients in the placebo group, 38.0% of patients in the macitentan 3 mg group, and 31.4% of patients in the macitentan 10 mg group. Worsening PAH was the most frequently documented endpoint regardless of whether patients were on background therapy. Although there was no significant difference in mortality among the three groups, there was significant improvement in secondary endpoints at 6 months (FC, exercise capacity, and hemodynamics). The major side effects noted with macitentan were headache, nasopharyngitis, and anemia; however, there were no differences in rates of transaminitis or edema. Based on the clinical efficacy and safety profile, the FDA approved macitentan 10 mg in 2013 as an oral therapy for WHO group 1 patients with FC II and III symptoms. Of note, a study published in 2015 showed that disease progression was reduced in the treatment-naïve cohort in SERAPHIN in both incident (diagnosis <6 months, n = 110) and prevalent (>6 months, n = 157) patients taking macitentan ^19^.

### Oral treprostinil

Although there is evidence that prostanoid therapy improves both morbidity and mortality in PAH, such therapy is under prescribed ^20^. As a result, the search for effective and well-tolerated routes of delivery for prostanoid therapy continues. The series of FREEDOM studies (FREEDOM-M, -C, and -C2) trialed the use of treprostinil diolamine, an oral form of the prostacyclin analogue treprostinil. In FREEDOM-M, a randomized, placebo-controlled, phase 3 study of 349 treatment-naïve PAH patients, there was significant improvement in 6MWD by 23 m at 12 weeks (95% confidence interval of 4 to 41 m, *P* = 0.0125) but no improvement in FC or TTCW ^21^. Based on these results, oral treprostinil was FDA approved as monotherapy in PAH patients in 2013. Oral treprostinil is contraindicated in patients with Child-Pugh stage 3 hepatic impairment because of hepatic metabolism of the drug and is discouraged for use in pregnant women ^22^.

In the first completed oral treprostinil study, FREEDOM-C, up-titration of the drug was limited by drug tolerance ^23^. In contrast, FREEDOM-M showed that divided low-dose administration, starting at 0.125 or 0.25 mg and increased gradually every 3 or 4 days, improved tolerance. This ultimately may aid in improved patient adherence and ability to prescribe the drug early in the disease course. Future directions from this study include investigations of whether the effects seen with escalating doses of intravenous (IV) prostanoid therapy are also seen with oral therapy, as well as assessing the efficacy of oral treprostinil compared with other oral therapies ^20^.

### Selexipag

As stated above, given the efficacy of prostanoid therapy, much focus has been placed on the development of oral medications that target this pathway. Selexipag is one such drug; it is a highly selective, high-affinity agonist of the prostacyclin receptor. In a placebo-controlled, phase 2 trial, selexipag was shown to increase cardiac index and significantly reduce PVR by 33% at week 17 in patients who were already receiving PAH treatment ^24^. The subsequent GRIPHON (Prostacyclin Receptor Agonist in PAH) study was an event-driven, multicenter, double-blind, placebo-controlled, phase 3 trial of 1,156 patients randomly assigned to selexipag or placebo ^25^. Patients eligible for enrollment included treatment-naïve patients and those who were receiving an ERA or PDE-5i (or both) at stable doses. Patients receiving prostanoid therapy were excluded. The primary endpoint, a composite of all-cause mortality and any PAH complication, occurred in 42% of patients in the placebo arm and 27% of patients in the selexipag arm (hazard ratio of 0.60, *P* <0.001). This effect was similar in the subgroups of treatment-naïve patients and patients on background PAH treatment, suggesting a potential for selexipag to be used as combination therapy with other currently available oral treatments. There was a significant reduction in 6MWD but no significant difference in mortality between the two study groups. Future directions from the GRIPHON study may include investigation for the efficacy of selexipag compared with parenteral prostanoid therapy, or its effects in patients with earlier disease course, as more than half of all patients in the study were classified as FC III or IV.

## New delivery modes of therapy

### Implantable intravascular delivery of treprostinil

Current methods for delivery of treprostinil include subcutaneous (SC) continuous infusion, IV continuous infusion, inhaled, and, now, oral. The highest doses achievable with treprostinil are with the SC or IV infusion. However, significant adverse events may be associated with both of these. The SC infusion is associated with significant site pain, whereas the IV administration via central venous catheters is associated with an increased risk for bloodstream infections and accidental interruption. To mitigate these events, a new, fully implantable intravascular delivery system for treprostinil infusion was investigated in the DelIVery trial ^26^. In this multicenter, prospective, single-arm clinical trial, an implantable drug delivery system was studied in 60 WHO group 1 PAH patients, with stable WHO FC I, II, or III symptoms, on a steady dose of IV treprostinil. System implantation consisted of the placement of a pump device in an abdominal pocket, which was connected to a tunneled catheter that inserted into the superior vena cava via a subclavian, cephalic, jugular, or axillary vein. At 6 months after implantation, there were no catheter-related bloodstream infections or catheter occlusions. Complications included those related to the implantation procedure, the catheter itself including catheter dislocation and venous stenosis, the pump e.g. pump pocket seroma, and the process of refilling the pump. The complication rate was 0.27 per 1,000 patient-days, and the 97.5% upper one-sided confidence bound (0.59 per 1,000 patient-days) was significantly less when compared with an objective performance criterion (2.5 complications per 1,000 patient-days). Additionally, there was a high rate of patient satisfaction with the implantable pump system, and WHO FC and 6MWD were maintained.

This study was limited by the absence of a parallel control group, and the authors noted a decrease in delivered drug past 6 months after implantation. As such, further investigation is needed to document the safety and accuracy of the delivery system prior to receiving FDA approval.

## Combination therapy

The AMBITION (Study of First-Line Ambrisentan and Tadalafil Combination Therapy in Subjects with PAH) trial was a multicenter, double-blind, phase 3 trial of 500 treatment-naïve WHO FC II and III PAH patients, randomly assigned to receive monotherapy with tadalafil (n = 121) or ambrisentan (n = 126) or both tadalafil and ambrisentan (n = 253) ^27^. The primary endpoint was time to first clinical failure, defined as death (all-cause mortality), hospitalization for worsening PAH, disease progression (>15% decline in 6MWD from baseline with FC III and IV symptoms), or an unsatisfactory clinical response (FC III symptoms while in the study for at least 6 months with a decrease in 6MWD from baseline). After a mean duration of 517 days, 18% of patients in the combination arm and 31% of patients in the pooled monotherapy arm met the primary endpoint event, demonstrating that upfront combination therapy in treatment-naïve patients resulted in a significantly lower risk of clinical failure events compared with monotherapy. The results were consistent across subgroup analyses of PAH etiology, WHO FC, age, gender, and geographical area. The difference was driven mainly by a marked reduction in hospitalization with combination therapy compared with the pooled monotherapy group (12% versus 4%). After 3 years, 68% of patients in the combination arm remained event-free compared with 56% in the pooled monotherapy group. Combination therapy was generally well tolerated. However, the combination group had a higher proportion of patients with edema (45% versus 30%), headaches (42% versus 34%), nasal congestion (21% versus 14%), and anemia (15% versus 9%). Among the secondary endpoints studied, three demonstrated outcomes in favor of the combination group. The change in 6MWD at 24 weeks was +49 m in the combination group compared with +27 m and +22 m in the ambrisentan and tadalafil monotherapy arms, respectively. In addition, NT-proBNP was significantly reduced in the combination therapy compared with the monotherapy subgroups. Finally, a higher percentage of patients with a satisfactory clinical response was observed in the combination group (39% versus 29%, odds ratio of 1.56, confidence interval of 1.05 to 2.32, *P* = 0.03). There was no difference in the change in WHO FC or Borg dyspnea scale between the groups.

Based on the results of the AMBITION trial, the 2015 European Society of Cardiology/European Respiratory Society (ESC/ERS) guidelines recommend that upfront combination therapy with tadalafil and ambrisentan should be offered to treatment-naïve WHO group 1 PAH patients with FC II or III symptoms as the first-line therapy ^2^. However, it must be stressed that the AMBITION study did not conclusively demonstrate that upfront therapy was superior to sequential therapy, as the study design did not include this arm. Another consideration is whether the benefit of upfront combination therapy is related to drug class rather than the choice of agents from each class. A retrospective study by Sitbon and colleagues documented the effect of upfront therapy with different combinations of PDE-5is (sildenafil and tadalafil) and ERAs (bosentan and ambrisentan) in newly diagnosed WHO group 1 PAH patients, 86% of whom were WHO FC III or IV on presentation ^28^. After 4 months, all regimens resulted in significant improvements in FC, exercise capacity, and hemodynamics with 1-, 2-, and 3-year survival rates that matched those predicted by the French equation. Despite the retrospective nature of the study, the data seem to support that any combination of PDE-5is and ERAs may be a viable option as first-line therapy for patients with newly diagnosed PAH, but this should be tested further in a randomized, blinded trial.

Finally, given the apparent benefits of initiating dual therapy in newly diagnosed patients, it is worth asking how an upfront triple-therapy approach incorporating prostanoids may perform in this setting. Despite their well-documented clinical efficacy, prostanoid therapies are associated with debilitating side effects, including headaches, nausea, and diarrhea, that could compound those of the other drug classes and disrupt quality of life. In a pilot study, 19 newly diagnosed FC III–IV WHO group 1 PAH patients were initiated on triple-therapy (IV epoprostenol, bosentan, and sildenafil); after 4 months, all patients tolerated the treatment and demonstrated significant improvements in exercise capacity, FC, and hemodynamics ^29^. A multicenter, double-blind, placebo-controlled trial to measure the efficacy and safety of upfront triple versus dual oral combination therapy—TRITON, NCT02558231—has recently started and hopefully will deliver more data on this question in the near future.

## Current treatment algorithm

**Figure 2.**
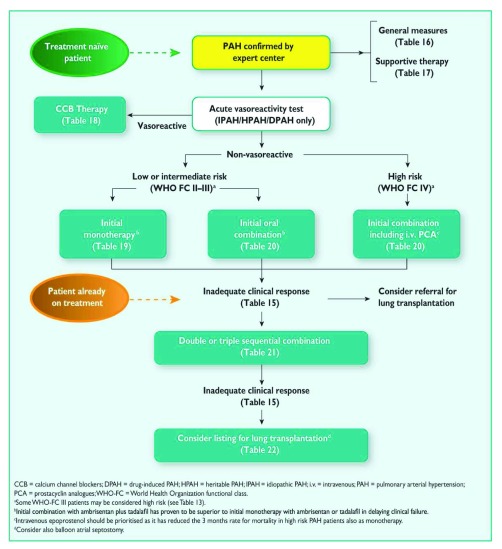
Treatment algorithm from the 2015 European Society of Cardiology/European Respiratory Society guidelines for the diagnosis and treatment of pulmonary hypertension. Reproduced with permission from the European Respiratory Society and European Society of Cardiology ^2^.

Figure 2 presents the current treatment algorithm included in the 2015 ESC/ERS joint statement. Once the diagnosis of WHO group 1 PAH is confirmed by right heart catheterization, initial stepwise or upfront combination therapy can be chosen for FC II or III patients, whereas FC IV patients should be offered parenteral prostanoids. Only the upfront combination of tadalafil and ambrisentan received class I recommendations (that is, evidence or consensus views or both support their benefits, usefulness, and effectiveness) owing to the lack of supporting evidence for other drug combinations. Advancement of therapy should be considered for patients with a high-risk profile, with the goal of achieving or maintaining a low-risk profile as outlined in Table 1. These parameters have a high predictive value and are strongly associated with survival outcomes after the initiation of PAH-targeted therapy. It is anticipated that this algorithm will continue to evolve as upcoming data on new treatment combinations and novel therapeutic agents become available.

**Table 1.** Risk assessment in pulmonary arterial hypertension, estimated 1-year mortality.

Determinants of prognosis	Low-risk <5%	Intermediate-risk 5%–10%	High-risk >10%
Clinical signs of right heart failure	Absent	Absent	Present
Progression of symptoms	No	Slow	Rapid
Syncope	No	Occasional	Repeated
WHO functional class	I, II	III	IV
6MWD	>440 m	165–440 m	<165 m
Cardiopulmonary exercise testing	Peak VO _2_ >15 mL/min per kg (>65% pred) VE/VCO _2_ slope <36	Peak VO _2_ 11–15 mL/min per kg (35–65% pred) VE/VCO _2_ slope 36–44.9	Peak VO _2_ <11 mL/min per kg <35% pred) VE/VCO _2_ slope >45
NT-proBNP plasma levels	BNP <50 ng/L NT-proBNP <300 ng/mL	BNP 50–300 ng/L NT-proBNP 300–1,400 ng/mL	BNP >300 ng/L NT-proBNP >1,400 ng/mL
Imaging (echocardiography and CMR imaging)	RA area <18 cm ^2^ No pericardial effusion	RA area 18–26 cm ^2^ No or minimal pericardial effusion	RA area >26 cm ^2^ Pericardial effusion
Hemodynamics	RAP <8 mmHg CI =2.5 L/min per m ^2^ SvO _2_ >65%	RAP 8–14 mmHg CI 2.0–2.4 L/min per m ^2^ SvO _2_ 60–65%	RAP >14 mmHg CI <2.0 L/min per m ^2^ SvO _2_ <60%

6MWD, 6-minute walk distance; BNP, brain natriuretic peptide; CI, cardiac index; CMR, cardiac magnetic resonance imaging; NT-proBNP, N-terminal pro-brain natriuretic peptide; pred, predicted; RA, right atrium; RAP, right atrial pressure; SvO _2_, mixed venous oxygen saturation; VE/VCO _2_, ventilatory equivalents for carbon dioxide; VO _2_, oxygen consumption; WHO, World Health Organization. Adapted with permission from the European Respiratory Society and European Society of Cardiology ^2^.

## The future: ongoing clinical trials

Treatment advances for PAH have consistently depended on the development of vasodilator agents targeting the pathways summarized in Figure 1. There is ongoing interest in determining whether the upfront combination of these agents holds greater benefit in PAH patients and whether single agents can be used to treat non-WHO group 1 PAH patients. However, most clinicians agree that the time is ripe for novel agents capable of targeting pathways relevant to the development and progression of PAH. Table 2 highlights ongoing studies with novel agents as well as representative studies focusing on combination and novel indications for available FDA-approved therapies.

**Table 2.** Current clinical trials in pulmonary hypertension.

Category of trial	Trial name (ClinicalTrials.gov identifier)	Trial description
Novel compounds	LIBERTY (NCT02736149)	Bestatin, a leukotriene A4 hydrolase antagonist, targeting leukotriene B4 production
	ASCO1 (NCT01086540)	Rituximab in systemic sclerosis-associated PAH (National Institutes of Health-sponsored)
	LARIAT (NCT02036970)	Bardoxolone methyl in PAH
	ARROW (NCT02234141)	GS-4997, an ASK-1 inhibitor, for use in PAH
	NCT02829034	Ranolazine for treatment of PAH
	NCT00964678	Carvedilol for treatment of PAH
Combination therapies	BEAT (NCT01908699)	Inhaled treprostinil with or without oral beraprost
	NCT02253394	Combination of ambrisentan and spironolactone in PAH
	TRITON (NCT02558231)	Efficacy and safety of initial triple versus dual oral combination therapy for PAH

ASK-1, apoptosis signal-regulating kinase 1; PAH, pulmonary arterial hypertension.

## Conclusions

Over the past 20 years, we have seen the arrival of 14 FDA-approved therapies to treat PAH, a major feat considering that this is a disease that affects a small segment of the population. The key to the success in developing PAH therapies has been the tremendous advances in understanding the genetic and molecular mechanisms that drive the pathogenesis of the disease. It is expected that, as the state of our knowledge grows, the treatment strategies will continue to evolve and diversify from the current paradigm centered on vasoconstriction. In the meantime, we will likely see data from trials exploring the clinical efficacy of combination therapies, novel delivery devices, and utility in other forms of pulmonary hypertension. With SERAPHIN, GRIPHON, and AMBITION, we have made a major leap forward in the design of clinical trials that will measure clinically relevant endpoints. This should be the standard for clinical trials in the future.
